# Transepithelial Fluid and Salt Re-Absorption Regulated by cGK2 Signals

**DOI:** 10.3390/ijms19030881

**Published:** 2018-03-16

**Authors:** Jianjun Chang, Yan Ding, Zhiyu Zhou, Hong-Guang Nie, Hong-Long Ji

**Affiliations:** 1 Institute of Metabolic Disease Research and Drug Development, China Medical University, Shenyang 110122, China; Jian.Chang@uthct.edu (J.C.); yding@cmu.edu.cn (Y.D.); 18842463125@163.com (Z.Z.); hgnie@cmu.edu.cn (H.-G.N.); 2 Department of Cellular and Molecular Biology, University of Texas Health Science Center at Tyler, Tyler, TX 75708, USA

**Keywords:** fluid and salt re-absorption, epithelial sodium channels, cystic fibrosis transmembrane conductance regulator, protein kinase, acute lung injury

## Abstract

Transepithelial fluid and salt re-absorption in epithelial tissues play an important role in fluid and salt homeostasis. In absorptive epithelium, fluid and salt flux is controlled by machinery mainly composed of epithelial sodium channels (ENaC), cystic fibrosis transmembrane conductance regulator (CFTR), Na^+^/H^+^ exchanger (NHE), aquaporin, and sodium potassium adenosine triphosphatase (Na^+^/K^+^-ATPase). Dysregulation of fluid and salt transport across epithelium contributes to the pathogenesis of many diseases, such as pulmonary edema and cystic fibrosis. Intracellular and extracellular signals, i.e., hormones and protein kinases, regulate fluid and salt turnover and resolution. Increasing evidence demonstrates that transepithelial fluid transport is regulated by cyclic guanosine monophosphate-dependent protein kinase (cGK) signals. cGK2 was originally identified and cloned from intestinal specimens, the presence of which has also been confirmed in the kidney and the lung. cGK2 regulates fluid and salt through ENaC, CFTR and NHE. Deficient cGK2 regulation of transepithelial ion transport was seen in acute lung injury, and cGK2 could be a novel druggable target to restore edematous disorder in epithelial tissues.

## 1. Introduction

Cyclic guanosine monophosphates-dependent protein kinases (cGKs) belong to the serine/threonine kinase family and are present in many eukaryotes ranging from unicellular organisms to human beings [1]. As the key enzymes in the downstream conduction pathway of cyclic guanosine monophosphates (cGMP), cGKs can be activated by gaseous NO and cytosolic cGMP signals. There are three cGK isoforms, cGK1α, cGK1β and cGK2. cGK1 has been detected at high concentrations in smooth muscles, platelets, cerebellum and other tissues [2]. Accumulating evidence suggests that cardiovascular functions are predominantly regulated by cGK1 [2,3]. Originally identified and cloned from intestinal specimens [4], expression of cGK2 has also been confirmed in kidney [5] and lung epithelial cells [6]. As a membrane-bound protein, cGK2 is mainly located in several cranial nuclei, small-intestinal mucosa, chondrocytes and pneumocytes. In recent years, researchers have been increasingly interested in the cGK2 isoform, and have deciphered some of its new functions [7]. Deficiency of cGK2 phenotype leads to dysfunction in epithelial tissues [8,9], impaired bone growth [10,11], and alternation in emotional behavior [12]. We here briefly review the progress of cGK2 on the regulation of fluid and salt balance in epithelial tissues.

## 2. Transepithelial Ion Transport Mechanism

**Figure 1:**
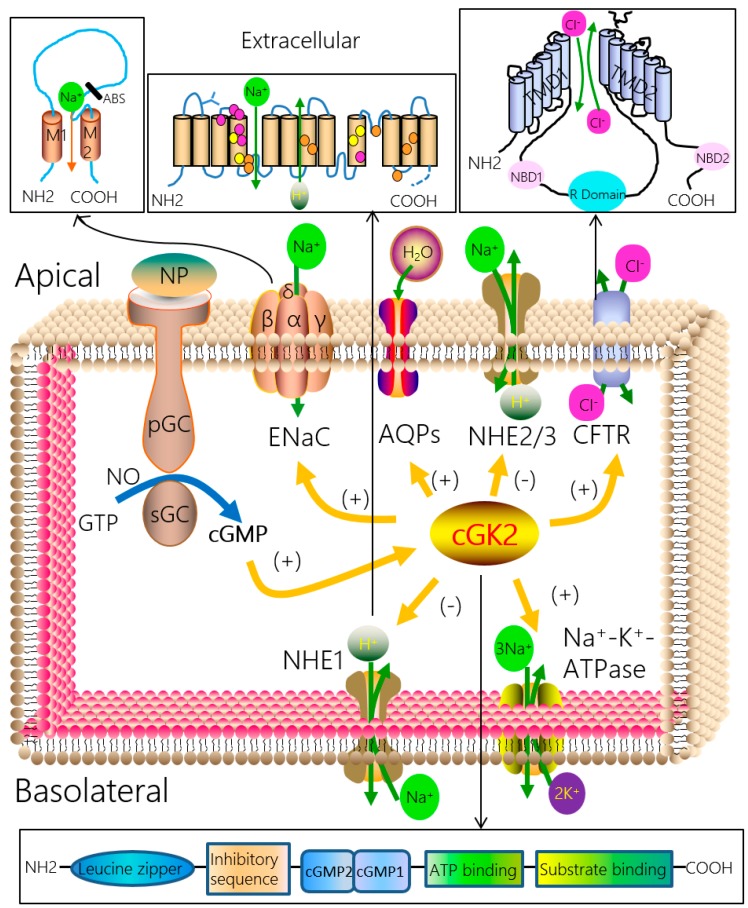
Regulation of fluid and salt transport by the cGK2 signal pathway in epithelial cells. Abbreviations: NP, natriuretic peptide; NO, nitric oxide; pGC, particulate guanylyl cyclase; sGC, soluble guanylyl cyclase; cGMP, cyclic guanosine monophosphates; GTP, guanosinetriphosphate; cGK2, cGMP-dependent protein kinase isoform 2; ENaC, epithelial sodium channel; M1 and M2, helical transmembrane domains; ABS, amiloride binding site; AQPs, aquaporins; CFTR, cystic fibrosis transmembrane conductance regulator; TMD1 and TMD2, transmembrane-spanning domains; NBD1 and NBD2, nucleotide-binding domains; R domain, regulatory domain; Na^+^-K^+^-ATPase, sodium potassium adenosine triphosphatase; NHE, Na^+^/H^+^ exchanger; pink circles, ion transport and inhibitor binding sites; orange circles, ion transport and binding sites; yellow circles, inhibitor binding sites; green arrows, direcction of transport; oriange arrows, direction of regulation; blue arrow, direction of reaction process; black arrows, direction of enlarged viewing.

The mechanisms of regulating active fluid and salt transport across the epithelium have become an area of research with vital implications for understanding fluid homeostasis under both normal and pathologic conditions. Epithelial fluid and salt transports are predominately controlled by the epithelial sodium channel (ENaC), cystic fibrosis transmembrane conductance regulator (CFTR), aquaporin, and Na^+^/K^+^-ATPase (Figure 1). The ENaC is located on the apical side of the epithelium and permeates sodium ions; for example, ENaC contributes to approximate 60% of transalveolar Na^+^ re-absorption in the lung. The CFTR is located in the apical membrane of epithelium, in addition to ENaC, it serves as a major route for the secretion of fluid in gut and may play a role in alveolar fluid clearance (AFC) [13]. In epithelium cells, only cGK2 is expressed, while two cGK1 isoforms are located in endothelial cells and excitable cells. Evidence regarding the regulation of transepithelial fluid and salt re-absorption by cGK2 signals is accumulating, in particular via ENaC and CFTR.

## 3. cGK2 Regulation of ENaC

Four mammalian subunits, α, β, γ, and δ ENaC, have been cloned so far. These transmembrane proteins are predominately expressed in the apical plasma membrane of epithelial cells in the lung, the kidney, the colon, and the airway, serving as a critical pathway for maintaining fluid/salt homeostasis locally and systematically.

Increasing evidence demonstrates the regulation of Na^+^-absorption by cGK2 in the small intestine, the nephron, and the lung [2,14]. We found that 8-(4-chlorophenylthio)-cGMP (8-pCPT-cGMP, a cGK2 activator) specifically stimulated αβγ-ENaC activity expressed in oocytes, whereas the cGK1 activator did not [15]. Furthermore, the transcripts of α-ENaC were increased by 8-pCPT-cGMP, probably by facilitating the expression of cGK2 at the mRNA level. Conversely, siRNA specific for cGK2 reduced the transcription of α-ENaC [16]. In addition to increased permeability through alveolar microvascular endothelium, lung edema usually results from reduced edema fluid resolution via ENaC [3]. AFC in vivo was improved by 8-pCPT-cGMP in mice [17]. Our study indicated that AFC increased significantly after administration of 8-pCPT-cGMP into human lungs intratracheally ex vivo. The potential mechanisms may be related to the elimination of self-inhibition of ENaC [18]. Moreover, the activation of cGK2 signals stimulated amiloride-sensitive short-currents across human alveolar epithelial cell monolayers and heterologously expressed αβγδ-ENaC activity in a dose-dependent manner. The activation of ENaC was inhibited by a specific cGK2 inhibitor [17]. Given the crucial role of ENaC in the resolution of lung edema, we examined the responses of ENaC to cGK2 signals in human pleural mesothelial cells, and found the up-regulation of ENaC by 8-pCPT-cGMP in Ussing chamber and whole-cell patch clamp experiments [19]. In addition to the lung, ENaC governs fluid and salt in the kidney. ENaC is responsible for renal sodium re-absorption and providing the driving force for potassium [3]. In this pathway, cGK2 has a pivotal role in the regulation of renal ENaC function and pathogenesis [20].

## 4. CFTR

CFTR is a cyclic adenosine-dependent chloride channel protein, the only ion channel in the adenosine triphosphate-binding cassette transporter family. Consisting of two membrane-spanning domains and a regulatory domain that regulates sodium channel, CFTR is expressed in many epithelial tissues, for example, the lung, the intestine, the pancreas and, the kidney [21]. The roles of CFTR include transepithelial movement of chloride ions, transportation of bicarbonate and glutathione, regulation of intracellular and extracellular fluid flowing and ion concentration, as well as transepithelial transportation of salts.

In rat intestine epithelium cell line, which stably expresses CFTR, some studies have demonstrated the effects of cGK2 on activating CFTR [8,22], while the inhibitors of cGK2 suppressed the activation of CFTR in intestine epithelium [23,24]. The inhibition of cGK2 signals effectively reduced 8-pCPT-cGMP and *Escherichia coli* heat-stable enterotoxin (STa, an enterotoxin that stimulates cGMP accumulation and intestinal fluid secretion)-induced trafficking of CFTR to the cell surface of villus enterocytes activation. In contrast, blocking of cyclic adenosine monophosphate (cAMP)/protein kinases A (PKA) signaling did not alter the cell surface levels of CFTR [25]. These results reveal an important role of cGK2 signals in STa-dependent trafficking of CFTR in the intestine. 8-pCPT-cGMP and STa increased CFTR relative short-circuit current in the small intestine of wild-type mice, whereas the above enhancement of CFTR was markedly reduced in cGK2-deficient mice [26,27]. To investigate the molecular basis for the cGK2 isotype specificity of CFTR, researchers expressed cGK2 or cGK1 mutants possessing different membrane binding properties by using adenoviral vectors in a CFTR-transfected intestinal cell line and found that the mutation of cGK2 N-terminal myristoylation site reduced cGK2 membrane binding and severely impaired cGK2 activation of CFTR [28]. A later study also proved this observation [24]. In addition to intestine epithelium, researchers also found the presence of both immunoreactive and functional CFTR in the alveolar epithelium [29,30]. The chromosome mutations of CFTR led to cystic fibrosis (CF); interestingly, the existence of a complex of CFTR-NHERF2-lysophosphatidic acids receptor2 in airway and gut epithelium was reported recently, which may provide new therapeutic interventions for CF [31]. In the lung, CF involves the exocrine glands, causing increased mucus in the airways and repeated bronchial infection, and then leading to pulmonary CF. In human alveolar epithelial cell lines, guanylin induced the activation of CFTR, and this effect was related to cGK2 signal pathway [6]. Moreover, cGK2, but not cGK1, phosphorylated CFTR immunoprecipitated from human alveolar epithelial cells in vitro [32].

## 5. Na^+^/H^+^ Exchanger (NHE)

NHE are integral plasma membrane proteins catalyzing the electroneutral exchange of extracellular sodium for intracellular protons with a stoichiometry of one for one. They exist as homodimers through intermolecular interactions, and their N-terminus contains 12 transmembrane domains that are involved in ion transportation [33]. Nine NHE isoforms have been discovered in mammalian cells, referred to as NHE1–NHE9, of which NHE1–NHE5 are expressed in the plasma membrane, and NHE6–NHE9 localize to the intracellular membranes [34]. NHE1 localizes to the basolateral membrane of various epithelial cells, while NHE2 and NHE3 are mainly found in the apical side of epithelial cells of kidney and small intestine [35,36]. As important transmembrane transporters, the multiple functions of NHE include the regulation of intracellular pH, the control of cell volume and transepithelial ion transportation.

cGK2 is located in the secretory epithelium of the kidney and the small intestine, and regulates the metabolism of sodium and protons, possibly through the mediation of NHE. The inhibition of cGK signaling abolishes the suppression of pH recovery induced by NHE inhibitors in renal tubular epithelial cells [37]. A recent study indicated that cGK signaling regulated NHE1 function by promoting the production of reactive oxygen species in renal epithelial cells [38]. Inhibition of NHE3 by 8-pCPT-cGMP was observed in the presence, but not absence, of cGK2 in vivo. Moreover, cGK2 bound to NHE regulatory factor (NHERF)2 in order to regulate NHE3 trafficking [39,40]. On the other hand, cGK2 directly phosphorylated NHE3 at three sites to suppress NHE3 activity [41]. In intestine epithelium, cGK2 increased Na^+^ absorption in the small intestine epithelium by inhibiting NHE3 [2,27].

## 6. Acute Lung Injury (ALI)

ALI is a common clinical condition caused by infectious and non-infectious insults, the current therapy for which is supportive, and there is an urgent need for novel and more effective interventions. AFC is the resolution of fluid by alveolar epithelium consisting of ENaC, aquaporin and Na^+^/K^+^-ATPase. As one of the characteristics of ALI during the early exudative phase, pulmonary edema results from the imbalance of AFC and turnover. An increasing number of studies have confirmed that cGK2 signals mediate the attenuation of ALI induced by lipopolysaccharide [42,43]. As a specific cGK2 activator, 8-pCPT-cGMP increases antioxidant function and attenuates oxidant cell death in ALI animal models [44,45]. Studies of transepithelial ion transport in lung have demonstrated that cGK2 regulated mice and human AFC, and that the underlying mechanisms may be related to the regulation of alveolar ENaC by cGK2 signals [17,18]. Regulating AFC by cGK2 signals may expedite the solution of pulmonary edema, which will provide a new and promising intervention to ALI.

## 7. cGK2 Signals in Drug Discovery

Several strategies have been applied in the development of cGK2-specific activators, which interact with the binding sites for cGK2 substrate peptides. The cGK2-specific activation drugs may be useful for the treatment of substantial pulmonary diseases by modulating the transport of fluid and salt. The membrane-permeable analogues can interact with cGK2 at the cGMP binding sites, which can be used as tools for the treatment of pulmonary diseases [46]. The most potent cGK2 activator, 8-pCPT-cGMP, is resistant to hydrolysis by phosphodiesterases. Studies on the effects of the cGK2 activator on pulmonary diseases have never halted. Earlier research demonstrated the role of the cGK2 activator in pulmonary hypertension [47] and lung transplantation [48] in animal models. In respiratory cells, 8-pCPT-cGMP was also able to alter transepithelial fluid and salt transport by up-regulating CFTR [49] or cation channels [18,50], which indicated the roles of the cGK2 activator in edematous lung injury.

Another activator associated with cGK2 signals is natriuretic peptide (NP). NP binds to the NP receptors that contain the intracellular guanylyl cyclase domain, which catalyzes guanosine triphosphate and produces cGMP [51]. Mainly secreted by atrial myocytes, the roles of atrial NP (ANP) have been confirmed in the activation of CFTR at transcription and protein levels in rat colon epithelium and human intestine epithelial cells [52]. In addition to CFTR, ANP also regulated ENaC activity in urinary bladder cells of Japanese tree frog [51]. C-type NP increased CFTR-associated chloride permeability by activating the cGMP/PKA signaling in both normal and CF airway epithelial cells [53,54]. Moreover, a recent study reported that a synthesized guanylyl cyclase 2C agonist resulted in the functional rescue of CFTR mutants in CF mice and patients [55]. Altogether, NP regulated transepithelial fluid and salt re-absorption partly through ENaC and CFTR. The activation of cGKs or/and PKA was required, due to the functional and pharmacological cross-talk between PKA and cGKs signaling pathways [56,57]. Acting as guanylyl cyclase activators, NP may regulate cGK2 signals through cAMP/PKA or cGMP/cGK2 signaling.

## 8. Conclusions and Perspectives

The metabolism of fluid and salt in epithelium is crucial for the occurrence and development of many diseases, such as pulmonary edema and CF. Researchers have been studying cGKs for many years, and evidence regarding the regulations of transepithelial fluid and salt re-absorption by cGK2 signals is accumulating, most of which focuses on the whole cGKs or cGK1. Studies regarding the effects of cGK2 on the metabolism of fluid and salt are limited, and this emerging field has not been synopsized to date. This review mainly summarized the increasing evidence regarding the effects of cGK2 signals on transepithelial fluid and salt transport and the underlying relative mechanisms. We can conclude that cGK2 signals regulate transepithelial fluid and salt re-absorption partly through the mediation of Na^+^, H^+^ and Cl^−^ by regulating ENaC, CFTR and NHE (Figure 1). In addition, we state the roles of cGK2 signals in ALI and the potential of drugs relative to cGK2 signals in the treatment of pulmonary diseases, both of which may provide a new and promising intervention to the occurrence and development of pulmonary diseases, such as ALI. In addition to important ENaC and CFTR in epithelial tissues, there are also other pathways for fluid and salt transport, such as aquaporin and Na^+^/K^+^-ATPase, which have roles in the metabolism of potassium ion and fluid. However, our understanding of the cellular and molecular mechanisms by which cGK2 signals regulate other transepithelial fluid and salt-metabolizing pathways are incomplete and limited. Future studies including greater awareness of these metabolism pathways and explaining their modulation by cGK2 signals will provide more evidence of the mechanisms by which cGK2 signals regulate the metabolism of fluid and salt, satisfying the urgent need for novel and more effective interventions in pulmonary diseases.
